# “We do not bury dead livestock like human beings”: Community behaviors and risk of Rift Valley Fever virus infection in Baringo County, Kenya

**DOI:** 10.1371/journal.pntd.0005582

**Published:** 2017-05-24

**Authors:** Edna N. Mutua, Salome A. Bukachi, Bernard K. Bett, Benson A. Estambale, Isaac K. Nyamongo

**Affiliations:** 1 Institute of Anthropology, Gender and African Studies, University of Nairobi, Nairobi, Kenya; 2 Animal and Human Health, International Livestock Research Institute, Nairobi, Kenya; 3 Research, Innovation and Outreach, Jaramogi Oginga Odinga University of Science and Technology, Bondo, Kenya; 4 Cooperative Development, Research and Innovation, The Cooperative University of Kenya, Nairobi, Kenya; School of Veterinary Medicine University of California Davis, UNITED STATES

## Abstract

**Background:**

Rift Valley Fever (RVF), is a viral zoonotic disease transmitted by *Aedes* and *Culex* mosquitoes. In Kenya, its occurrence is associated with increased rains. In Baringo County, RVF was first reported in 2006–2007 resulting in 85 human cases and 5 human deaths, besides livestock losses and livelihood disruptions. This study sought to investigate the county’s current RVF risk status.

**Methodology and principal findings:**

A cross-sectional study on the knowledge, attitudes and practices of RVF was conducted through a mixed methods approach utilizing a questionnaire survey (n = 560) and 26 focus group discussions (n = 231). Results indicate that study participants had little knowledge of RVF causes, its signs and symptoms and transmission mechanisms to humans and livestock. However, most of them indicated that a person could be infected with zoonotic diseases through consumption of meat (79.2%) and milk (73.7%) or contact with blood (40%) from sick animals. There was a statistically significant relationship between being male and milking sick animals, consumption of milk from sick animals, consuming raw or cooked blood, slaughtering sick livestock or dead animals for consumption (all at p≤0.001), and handling sick livestock with bare hands (p = 0.025) with more men than women engaging in the risky practices. Only a few respondents relied on trained personnel or local experts to inspect meat for safety of consumption every time they slaughtered an animal at home. Sick livestock were treated using conventional and herbal medicines often without consulting veterinary officers.

**Conclusions:**

Communities in Baringo County engage in behaviour that may increase their risk to RVF infections during an outbreak. The authors recommend community education to improve their response during outbreaks.

## Author summary

The study focuses on the knowledge and socio-cultural practices around Rift Valley Fever (RVF) in Baringo County. It is intended to identify means through which communities in Baringo County could be exposed to RVF in the event of an outbreak. Specifically, it addresses knowledge of RVF transmission routes, practices in handling and consumption of meat, milk and blood; livestock disease management and disposal of dead animals/aborted foetuses. The study found that community members engaged in practices that would expose them to RVF in the event of an outbreak. These practices include milking and consuming milk from sick animals; consuming meat from slaughtered sick animals and those that die from disease; rarely having animals that were slaughtered at home inspected by a veterinary officer or a local animal expert before consumption; using uncertified techniques to test meat for safety of consumption; and treating sick livestock with both conventional and herbal treatments without the guidance of veterinary personnel. Further, RVF infections are likely to follow a gendered pattern based on the division of labor in livestock production. Based on their results, the study authors recommend community education to increase RVF awareness.

## Introduction

Rift Valley Fever (RVF) is a zoonotic disease associated with human and livestock morbidity and mortality as well as decreased trade in livestock and derived products. It is a viral disease caused by a *Phlebovirus* of the *Bunyaviridae* family [1–3]. It is transmitted by infected aedine and culicine mosquitoes [4, 5] and through contact with infected animal tissue and secretions [1, 2]. To date, there is no evidence of human to human RVF transmission [6]. Domestic ruminants, mainly cattle, sheep and goats are susceptible to RVF [3]. Infection with the RVF virus causes distinct disease in animals and humans. In livestock, the disease is marked by mass abortions in pregnant animals and mortality in newborns [1]. In humans, RVF often manifests as a mild febrile illness that may go undetected [7]. In rare occasions, the infections develop into severe disease causing hemorrhage, encephalitis and fatalities in 1% of cases and ocular impairment in 0.5–2% [7]. Due to its public health and economic impacts, RVF is categorized as “notifiable” by the Kenya government, thereby requiring that all suspected livestock and human cases within Kenya be reported to the government, which upon confirmation must formally inform the World Organization for Animal Health (OIE) [8] and the World Health Organization (WHO)[9], respectively.

Initially, RVF outbreaks were spatially confined to Africa (including Madagascar) but in the year 2000, the disease spread to the Arabian Peninsula [10]. In East and South Africa, RVF outbreaks are associated with wet seasons with higher than normal rainfall resulting in floods [3, 11, 12] which subsequently encourage multiplication of RVF vectors [1, 5]. However, RVF can occur in the absence of rain as has been witnessed in North and West Africa where it is linked to increased mosquito populations in large rivers and dams [3]. Movement of infected vectors, persons and animals could also lead to emergence of the disease in non-endemic areas [13].

In Kenya, RVF was first characterized in 1931 [3], and has since been reported nine more times with the latest outbreak in 2006 [6]. The outbreaks have mainly occurred in northern Kenya, mainly in Ijara and Garissa areas [11,13–15]. The 2006–2007 outbreak is estimated to have cost the country US$32 million (1US$ = 65Kenya shillings) in losses of livestock, livestock productivity, trade in livestock and livestock products and allied services [14]. Of the 340 confirmed human cases of RVF in Kenya during the 2006–2007 outbreak 60% were from the northern regions including Garissa (31%), Ijara (22%) and Wajir (5%) areas [15]. A further 10% were from the coastal district of Kilifi [15]. Occurrence of RVF in Baringo County was first recorded during the 2006–2007 outbreak [15]. The outbreak occurred against the backdrop of high cattle, sheep and goat populations in the County [16] and flooding in the lowland areas around lake Baringo following the exceptionally heavy rains of 2006 [17]. The affected areas also have solanchak soils which have previously been linked to RVF in Northern Kenya [17].

The most effective method of controlling RVF in Kenya is livestock vaccination but it is done inconsistently due to irregular outbreaks [11, 18]. Further, delays in laboratory confirmation of RVF cases result in ineffective and untimely corrective interventions [17, 19]. Bans placed on livestock trading and consumption of derived products during outbreaks for disease control are not only difficult to enforce but also pose great dietary and livelihood challenges to communities [1, 20]. Most human RVF cases have been attributed more to risky handling or consumption of livestock and derived products than bites from infected vectors as exemplified in South Africa [21], Mayotte [22], Tanzania [23], West Africa [24] and Kenya [1, 15, 25].

Previous social studies on RVF in Kenya have focused mainly on Northeastern Kenya where most RVF outbreaks have occurred [14, 26–28]. In Baringo County, RVF studies have focused on RVF vectors [29, 30], and human [15] and livestock serology [17]. No studies have been done to determine how knowledge and socio-cultural practices influence community risk to RVF in the area. This paper generates additional information on the role of knowledge, attitudes and practices in the transmission dynamics of RVF in Baringo County. It also explores differences in risk to RVF infection, between men and women and among zones.

## Materials and methods

### Ethical statement

The study acquired both national and the World Health Organization (WHO) ethical clearance referenced P70/02/2013 and Protocol ID B20278 respectively. All participants were of consenting age (18 years and above) and were required to give written consent before engaging in any research activity. For illiterate participants, the researcher read out the consent form details and allowed participants to consent through provision of a thumb print instead of a signature.

### Study area

**Fig 1:**
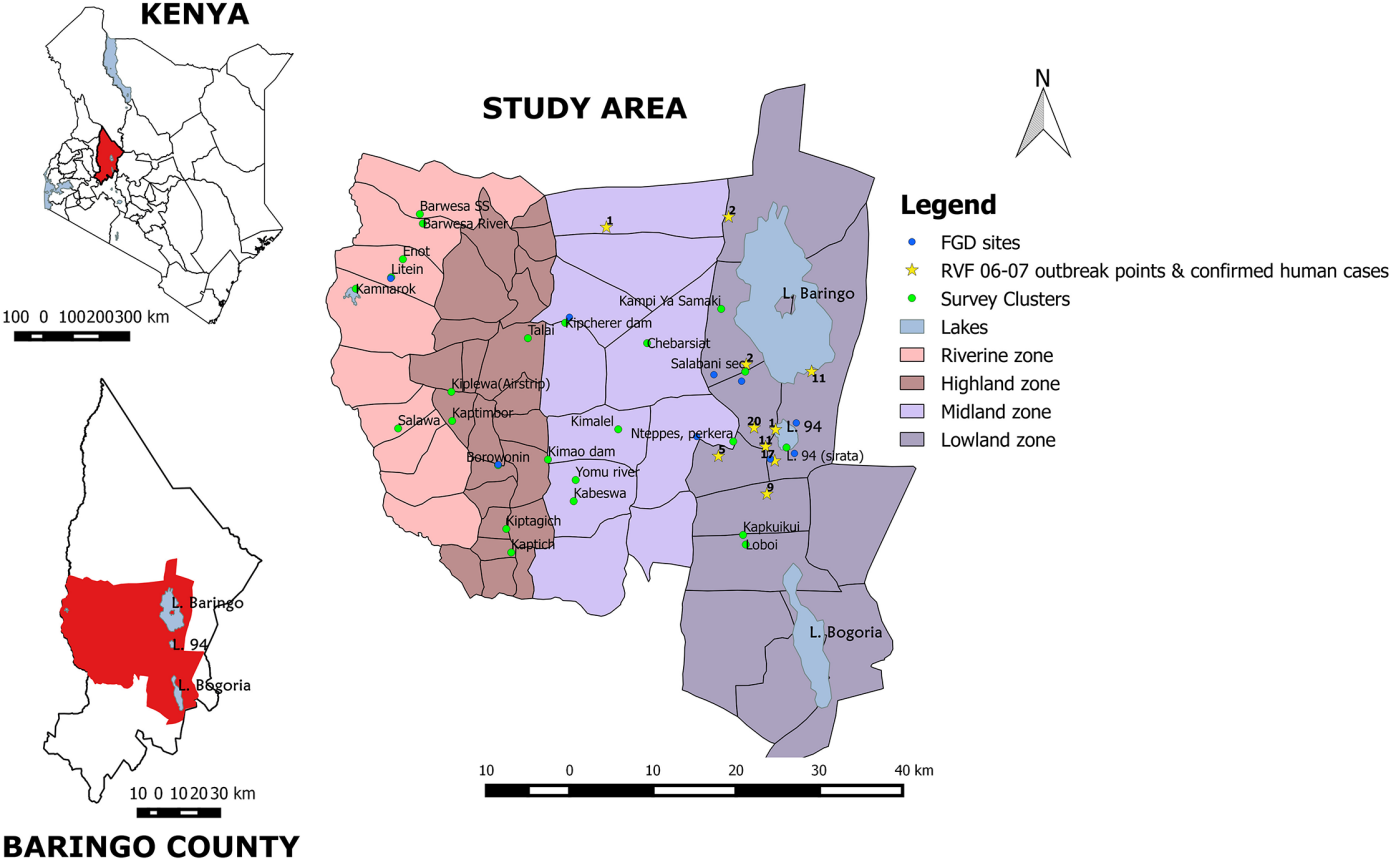
Map of Baringo County showing the study site.

The study took place in Baringo County’s Central, North and Marigat sub-counties. The research team classified the study site into four zones namely the highland, midland, lowland and riverine based on altitude. The highland has an altitude of >1500m above sea level (asl), midland >1000m-1500m asl, the lowland and riverine zones at <1000m asl. (Fig 1). The riverine zone is the area on the extreme left while the lowland zone is on the extreme right of the study site map, (Fig 1). The Tugen, a sub-tribe of the Kalenjin community, mainly inhabit the highland, midland and riverine zones while the Ilchamus who are a sub-tribe of the Maa community, are found in the lowlands. Both communities practice agriculture but the Tugen engaged in both crop and livestock farming while the Ilchamus are mainly livestock keepers. The 2006–2007 RVF outbreak occurred only in the lowland zone where the Ilchamus are found.

### Study design, sampling and data collection

The study utilized a cross-sectional research design in which a Knowledge, Attitudes and Practices (KAP) survey and focus group discussions (FGDs) were conducted sequentially. Survey respondents and focus group discussants were mutually exclusive. Qualitative data on livestock production and livestock disease management practices was collected first and comprised of 26 FGDs. Due to differences in zones, sex and community distribution, iterations resulted in 26 FGDs (13 male only and 13 female only) with a total of 231 discussants. For triangulation of male and female views, four FGDs were conducted per zone, in the highland, midland and riverine areas among the Tugen. In the lowland zone, 10 focus group discussion were conducted among the Ilchamus and 4 in a rural town that had mixed communities. Purposive sampling technique was used to select FGD discussants. To qualify as a discussant, an individual had to be 18 years old and above, have lived in the area for at least one year, be a current livestock keeper or from a livestock keeping household or consumer of livestock products with previous experience in livestock keeping.

The KAP survey, whose questionnaire was informed by the FGD’s findings, targeted 560 individuals drawn from the four ecological zones. The sample size was determined through the proportion to size sampling methodology for a finite population which resulted in 383 respondents (from 20 clusters, 5 clusters per zone). A further 5% was added to cover for possible incomplete questionnaires resulting in a sample of 400 (rounded figure). Owing to a desire to increase the external validity of the survey findings, the researchers proportionately increased the sample size by 8 respondents per cluster leading to a total sample size of 560. In each zone, clusters were selected from areas with at least 30 households. Thereafter, survey respondents were identified through simple random sampling ensuring that both men and women were proportionately represented. The zoning of the study site was used to assess whether there were any differences in knowledge and practices on livestock keeping and handling of animal products.

The FGD guide was pretested in 2 separate FGDs comprising exclusively of men or women to check for its suitability. Similarly, the survey questionnaire was pretested with 40 respondents in three areas which shared similar characteristics with the sampled sites. These sites were consequently excluded from the main survey. Final adjustments were made to the FGD and survey tools prior to data collection. Only Tugen and Ilchamus speaking enumerators participated in data collection.

### Data management and analysis

Survey data was analyzed in SPSS version 23 (IBM SPSS Statistics, Armonk, New York) after importation from CSPRO version 6.1 (United States Census Bureau, Washington DC) where it was entered and cleaned. Besides summary statistics, independent t-tests, one way ANOVA and Chi square tests were conducted to determine the relationship between different variables. Missing values were excluded from the analyses. The measure of statistical significance for this study was set at a p-value of 0.05. A binary logistic regression was also fitted to assess the association between respondents’ level of risk to RVF infection and demographic characteristics which comprised of zone of residence, sex, age, education level, marital status, household headship type, number of children, individual scores of knowledge of RVF transmission modes and livestock (cattle, sheep and goats) quantities measured in Tropical Livestock Units (TLUs) as guided by Cholinda and Otte [31]. The overall knowledge of possible RVF transmission routes was determined through eight questions on contact with sick animals, animal tissue, secretions and consumption of products from sick animals in the KAP survey. Each question had a Likert scale type of response where those who wholly disagreed, somewhat disagreed or neither agreed nor disagreed with a possible RVF transmission route were classified as not knowledgeable and scored a zero while those who somewhat or wholly agreed were classified as being knowledgeable and awarded a score of 1. The eight answers that a respondent gave were used to generate a cumulative score on knowledge ranging from 0–8.

The level of risk of exposure to the RVF virus was determined through 23 KAP survey questions with Likert scale responses ranging from never engaging in a given practice, or engaging very few times, sometimes, most of the times or always. The questions addressed community practices on: handling and consumption of milk, meat and blood; disposal of dead livestock; management of animals that abort; foetus disposal; and handling and treatment of sick livestock. For each question, respondents that carried out good practice were awarded a score of 1 while those that did not got 0. Individual outcomes were summed to give the total score per respondent. Respondents with scores below or equal to the mean were classified as high risk and above as low risk. The binary categorization of risk was used as the dependent variable in a binary logistic regression where the high risk category was coded as 0 and the low risk as 1. The model’s goodness of fit was tested using the Hosmer-Lemeshow test (χ^2^ = 0.617, df = 8, p = 1.000) and the omnibus-corpus test (χ^2^ = 180.799, df = 21, p<0.001) owing to the contested credibility of the Hosmer-Lemeshow test [32].

In each FGD, data was captured through note taking and audio recording. Audio files from the Tugen and Ilchamus were later transcribed directly verbatim into English by native speakers fluent in English and Swahili. Each script was verified through comparison of content with its recorded audio file and corresponding notes. Cleaned FGD data was coded into salient themes in Nvivo 10 (QSR international, Melbourne) and analyzed using the content analysis method. The emergent themes are presented together with the survey data in the results section.

## Results

### Respondent demographic characteristics

The KAP survey respondents (total of N = 560), n = 266 (47.5%) were male and n = 294 (52.5%) were female. Their average age was 44 years but most (n = 147, 26.3%) were aged between 27–35 years. Slightly more than half (n = 291, 52%) had primary education and were religiously affiliated to the Christian faith (n = 554, 99%). Most respondents (n = 439, 78.4%) were in monogamous unions while the others were either in polygamous unions (n = 59, 10.5%) or single (n = 62, 11.1%). Their main income sources were crop farming (n = 266, 47.5%) and livestock farming (n = 113, 20.2%). A total of 112 men and 119 women aged between 18–84 years with an average age of 41.7 years participated in the FGDs.

### Uses of livestock and derived products

In Baringo County, focus group discussants reported that livestock were considered stores of wealth; sources of food, medicine, income, manure, skins/hides, draft power, bride price, social status/prestige; and an indigenous means of predicting rainfall patterns (by “reading a goat’s stomach”) and conducting rituals. The main foods derived from livestock were meat, milk, blood, eggs and animal fat. However, meat and milk constituted a greater part of the communities’ diets compared to blood, eggs and animal fat. Among people suffering or recovering from diseases locally assumed to be severe, meat stock and milk were also used in the administration of conventional and herbal medicines. For children, medicines were ingested in or with milk whereas stock derived from boiling meat was favored for adults. Extracts from goat rumen and intestines believed to be medicinal were also used as reported by a male focus group discussants from Perkerra (lowland zone) and Borowonin (highland zone) saying, ““*eyande*” *[a green liquid extracted from the rumen of a goat] treats chronic malaria*,*”* and *“you get uncleaned goat small intestines*, *cut them into pieces*, *mix with herbs and boil*. *When cooked*, *you take them and become well*.*”* In the region, goat meat was most preferred, followed by beef then mutton. Discussants estimated local livestock proportions by species through a proportion piling exercise in which the moderator gave them 100 stones representing the total livestock population in their locality and asked to divide them proportionately to the livestock species they kept. The FGD participants estimated livestock populations at a median percentage of cattle 22.5% (range 13%-55%), goats 34% (range 5%-44%), sheep 20.5% (range 10%-30%), chicken 18% (range 7%-39%), donkeys 3.5% (range 0–10%), rabbits 0 (range 0–9%) and pigs 0 (0–8%).

### Knowledge of RVF signs and symptoms

**Fig 2:**
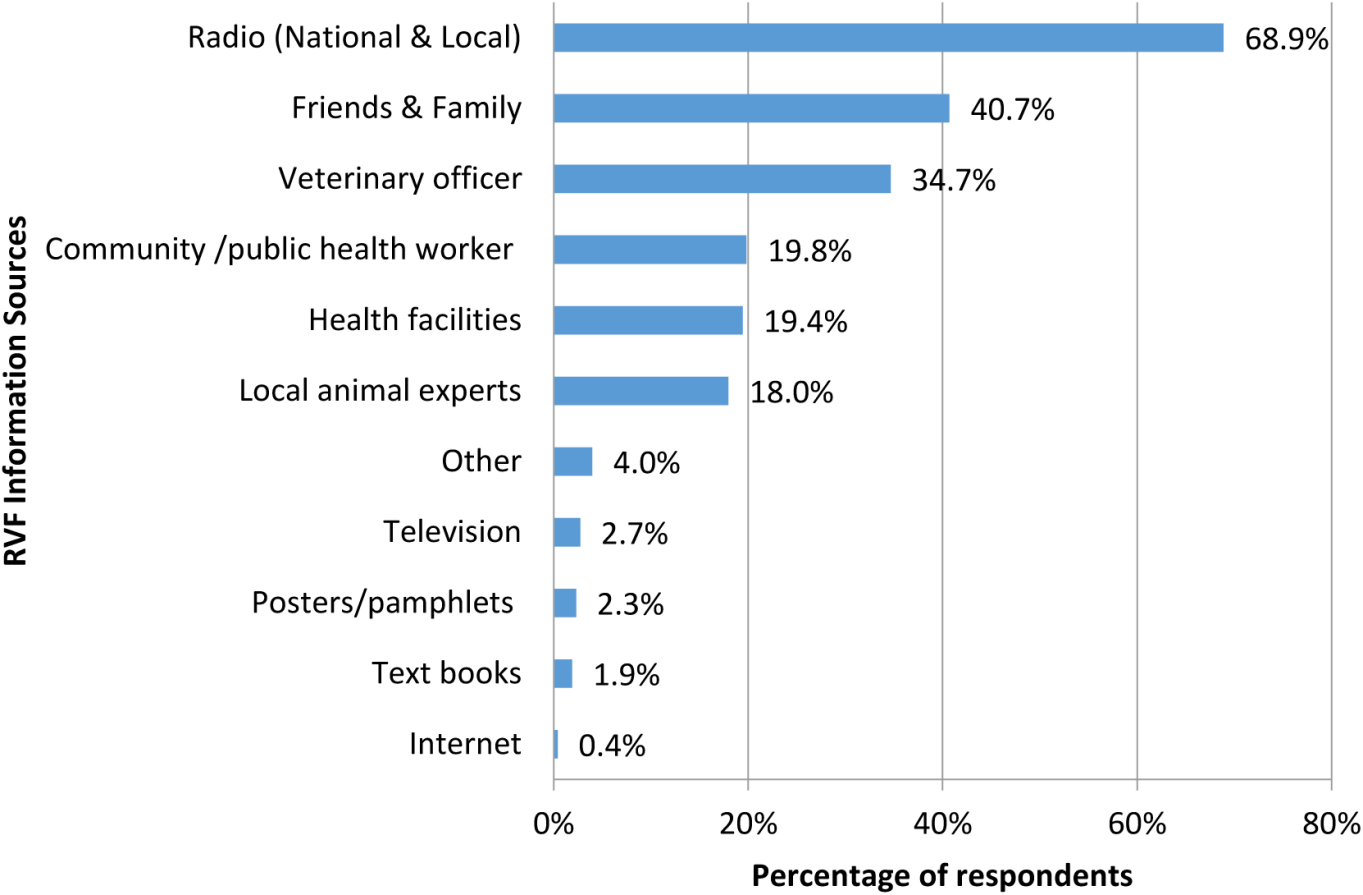
Sources of RVF information (multiple answers).

Based on the KAP survey data, n = 481 or 86% of the respondents had heard of RVF (N = 560). Among those who had heard of RVF (n = 481), the main sources of information on RVF were radio (n = 330, 68.6%), friends and family (n = 195, 40.5%), veterinary officers (n = 166, 34.5%), community/public health officials (n = 95, 19.8%), health facilities (n = 93, 19.4%) and local animal experts (n = 86, 17.9%) as shown in Fig 2. The least utilized sources were internet (n = 2, 0.4%), text books (n = 9, 1.87%), posters/pamphlets (n = 11, 2.29%) and television (n = 13, 2.7%). According to focus group discussants from the lowland zone where the 2006–2007 outbreak occurred, there were attempts to name the disease without consensus. Proposed names were *“the El -Nino livestock disease”* coined from the period when the disease last occurred and “*ngea na nyori*” which translated to “the greenish/yellow pigment” found in cadavers. Among the Tugen, the term *“kipkoloswo”* which refers to yellowing characteristic of those infected with yellow fever was also used to describe RVF disease in humans.

Few KAP survey respondents were knowledgeable of RVF signs and symptoms in humans (N = 559, excluding missing values). The following signs and symptoms were identified: fever (n = 85, 17.7%), headaches (n = 78, 16.2%), jaundice (n = 69, 14.3%), vomiting (n = 66, 13.7%), diarrhea (n = 61, 12.7%), bleeding from body openings (n = 56, 11.6%), joint pains (n = 54, 11.2%) and impaired vision (n = 40, 8.3%). A male focus group discussant from Lorok in the lowland zone reported that *“people infected with RVF showed some signs which resembled those of malaria; that is having very high fever*, *weak joints and having a headache”*.

### Knowledge of RFV causes and transmission routes

**Fig 3:**
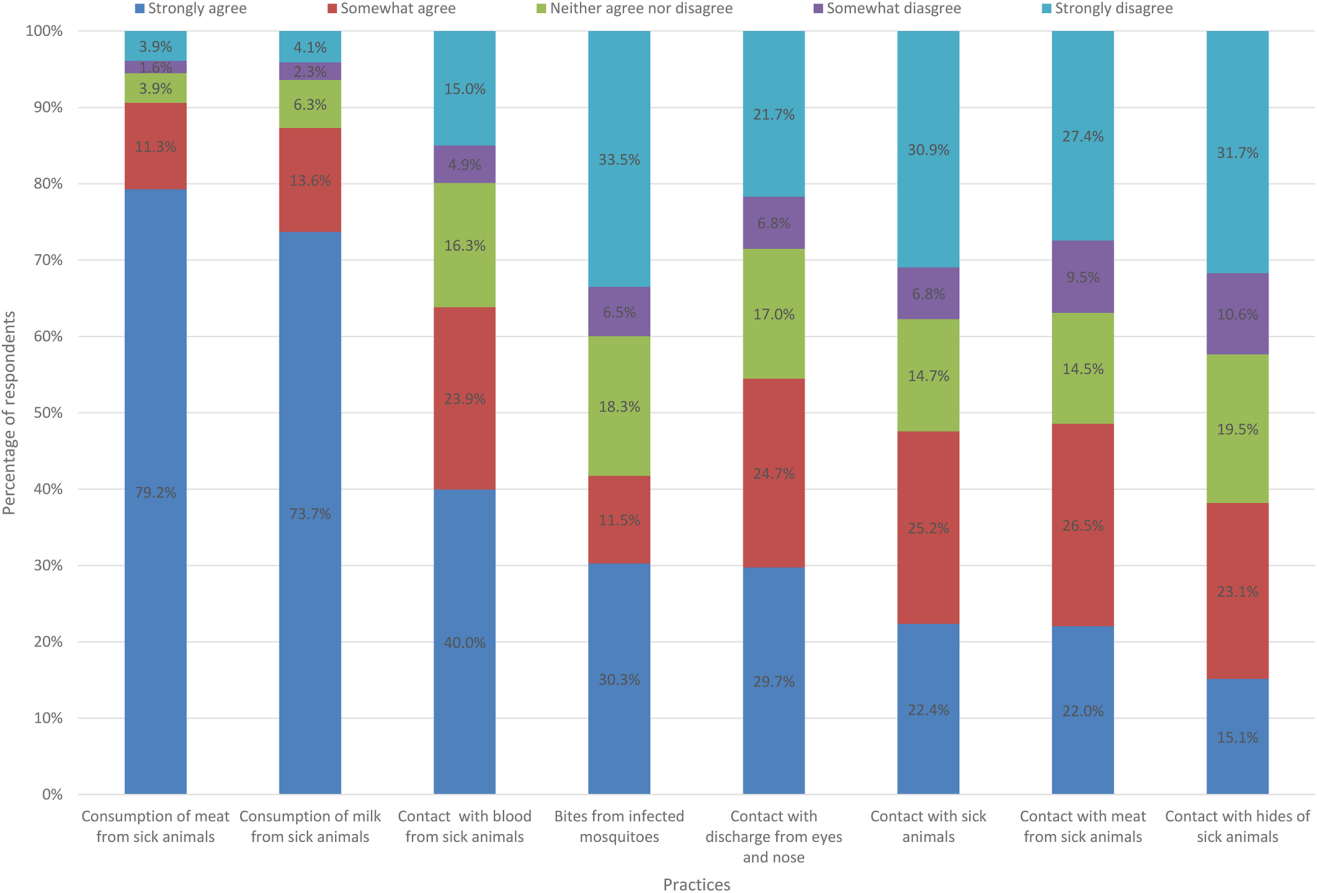
Communities’ knowledge of RVF exposure routes.

Survey respondents (N = 558) had limited knowledge of the cause of RVF. Only a third, n = 169 (30.3%) of respondents knew that mosquitoes had capacity to transmit a livestock disease to humans (Fig 3). Focus group discussants further reinforced this by implicating bad air, tsetse flies, ticks, monkeys and rains as causes of RVF. The main means through which KAP respondents believed they could be infected a with livestock zoonotic disease were through consumption of meat n = 442 (79.2%, N = 558) and milk n = 411, (73.7%, N = 558) or contact with blood n = 221, (40%, N = 553) from sick animals (Fig 3). Contact with sick animals n = 125, (22.4%, N = 559), their discharge from eyes and nose n = 166, (29.7%, N = 558), meat n = 123, (22%, N = 555), and skins/hides n = 84, (15.1% N = 555) were least associated with exposure to disease. Independents t-tests also showed that men and women had near equal mean knowledge scores of 4.54 and 4.84 respectively. There was no statistical difference between their knowledge levels *t*(558) = -1.383, p = 0. 167. Results from a one way ANOVA test showed that there were statistically significant differences between zones (F (3,556) = 6.571, p<0.001). Turkeys’ post hoc test further showed that there were statistically significant differences in knowledge score between the lowland and the midland (p = 0.001) and riverine zones (p = 0.003) but not the highland (p = 0.581).

### Regression of risk level and demographic characteristics

The mean and median scores of the 23 questions used to determine the level or risk of exposure to RVF was 12 while the lowest score recorded was 2 and highest 20. Those that had a score of ≤12 n = 326 (58.3%) were categorized as high risk and those with 13–23 n = 233 (41.7%) as low risk (N = 559). The binary categorization of risk scores was used as the grouping variable in a binary regression model fitted to test the association between level of risk of exposure to the RVF virus and demographic characteristics. Of the variables, the highland zone, male sex and scores on knowledge of RVF transmission routes were found to be statistically significant (Table 1). People from the highland zone had 9.253 times less risk of exposure to the RVF virus compared to those from the riverine zone. The odds of men engaging in unsafe practices were 0.176 times more that of women. The odds of engaging in safe practices were higher with increased respondents’ level of knowledge of RVF transmission routes. Specifically, an increase of 1 mean score in knowledge of RVF transmission routes significantly increased the odds of engaging in safe practices by 1.144.

**Table 1:** Regression on risk level and demographic characteristics.

Variables	P values	Odds Ratio	95% C. I. for Odds Ratio Lower-Upper
Highland	**<0.001**	**9.253**	**4.884–17.527**
Midland	0.806	0.927	0.506–1.699
Lowland	0.857	1.058	0.572–1.959
Riverine*	_	_	_
Male	**<0.001**	**0.176**	**0.106–0.293**
Female*	_	_	_
Single, never married	0.246	2.444	0.54–11.059
Married monogamous	0.281	1.935	0.583–6.421
Married polygamous	0.624	0.714	0.185–2.75
Separated	0.493	0.5	0.069–3.634
Widowed*	_	_	_
Male headed, with spouse	0.809	1.129	0.422–3.025
Male headed, no spouse	0.842	0.877	0.243–3.17
Female headed, no spouse*	_	_	_
Age in years	0.921	1.001	0.984–1.019
Children in household	0.394	0.958	0.867–1.058
No education	0.437	0.65	0.219–1.925
Primary education	0.992	0.995	0.403–2.461
Secondary education	0.733	1.178	0.459–3.023
Tertiary education	_	_	_
Crop farming	0.351	0.633	0.242–1.656
Livestock farming	0.08	0.395	0.14–1.116
Self-employment	0.299	0.58	0.208–1.621
Wage employment	0.465	0.646	0.199–2.09
Salaried employment	_	_	_
Total score on knowledge of possible RVF transmission routes	**0.007**	**1.144**	**1.037–1.263**
Livestock total TLUs	0.638	0.996	0.98–1.012
Constant	0.42	0.502	

*Reference group; bolded p values are statistically significant

Comparisons of mean scores on level of engagement in RVF risk practices between men and women through an independent T-test further confirmed that women engaged less in risk practices compared to men *t*(555) = -8.082, p<0.001. Women had a mean score of 12.87 while men had 10.45. By zone, mean scores showed that people in the highland zone engaged in less risk practices (14.40), followed by the midland (11.20), riverine (10.79) then lowland (10.22) in increasing order. Higher scores indicated low risk of exposure to the RVF virus and vice versa. Turkey’s post hoc tests, also showed that there was a significantly statistical difference between the highland and the other zones (p<0.001).

### Practices in relation to consumption of animal products

Majority of KAP survey respondents n = 508 (91.5%), reported that they always boiled their milk before consumption (N = 555). However, n = 349 (62.4%) never consumed milk from or milked n = 326 (58.7%) sick livestock. There was a statistically significant relationship between sex and milking (χ^2^ = 22.146, df = 4, p<0.001) and consumption of milk from sick animals (χ^2^ = 53.875, df = 4, p<0.001) (Table 2). Women, whose role it was to milk, were less inclined to milk sick livestock while men showed higher tendency to consume milk from sick livestock. When animals were slaughtered at home, n = 349 (62.7%, N = 556) of respondents reported that some household members had ever consumed the raw blood while n = 424 (76.3%, N = 556) had ever consumed it cooked. The association between sex and consuming raw (χ^2^ = 23.970, df = 4, p<0.001) or cooked blood (χ^2^ = 23.556, df = 4, p<0.001) was statistically significant (Table 2). A higher proportion of men, whose role it was to slaughter livestock, consumed raw or cooked blood more often than women.

**Table 2:** Statistical relationships between sex and livestock management factors.

	Practice or Perception	Statistical association with sex
1	Milking sick animals	(χ^2^ = 22.146, df = 4, p<0.001)
2	Consumption of milk from sick animals	(χ^2^ = 53.875, df = 4, p<0.001)
3	Consuming raw blood	(χ^2^ = 23.970, df = 4, p<0.001)
4	Consuming cooked blood	(χ^2^ = 23.556, df = 4, p = 0.001)
5	Slaughtering sick livestock for consumption	(χ^2^ = 50.909, df = 4, p<0.001)
6	Slaughtering dead animals for consumption	(χ^2^ = 11.358, df = 4, p<0.001)
7	Handling sick livestock with bare hands	(χ^2^ = 11.185, df = 4, p = 0.025)
8	Perception that veterinary services were expensive	(χ^2^ = 13.210, df = 4, p = 0.010)
9	Perception that accessing veterinary medicines was difficult	(χ^2^ = 32.627, df = 4, p<0.001)
10	Seeking veterinary services	(χ^2^ = 17.539, df = 4, p = 0.002)
11	Perception that veterinary services were easy to access	(χ^2^ = 33.915, df = 4, p<0.001)
12	Perception that veterinary medicines were difficult to administer	(χ^2^ = 26.884, df = 4, p<0.001)

More than half n = 331 (59.7%, N = 554), of the respondents had ever eaten meat from a sick animal that had been slaughtered. A further n = 282 (50.8%, N = 555) had ever eaten meat from an animal that died from sickness. There was a statistically significant relationship between sex and slaughtering sick livestock (χ^2^ = 50.909, df = 4, p<0.001) or dead (χ^2^ = 50.358, df = 4, p<0.001) animals for consumption with higher proportions of men than women likely to engage in both practices (Table 2). Only n = 131 (23.6%, N = 556) of respondents relied on trained personnel or local experts (n = 65, 11.7%, N = 555), to check the meat for safety of consumption every time they slaughtered. Focus group discussants reported that they applied other traditional methods besides utilizing services from the experts. These included observing the health reactions of those who consumed the meat earlier and if no harm occurred they would also consume of it as exemplified in the following excerpt.

*“There was a cow…*. *that they slaughtered*. *Some people took the meat but did not eat it immediately*. *They waited for other people to eat first so that if they [those who ate first] were affected the others [those who had not eaten] would not eat”*. Female discussant, Borowonin-1, highland zone

From various group discussions, different tree species used to cure meat slaughtered from sick/dead animals were identified. The tree species included *“soget”/”sokonyi” (Warbugia ugandensis)*, *“sessiat” (Acacia tortilis)*, *“subeiwa”/“ntepes” (Acacia nubica)*, and *“segetet” (Myrisine africana)*. Once meat was boiled with herbs from these tree species it was considered safe for consumption. Meat prepared in this manner was sometimes only consumed by a segment of the population. For instance, *“for an animal with anthrax*, *men exclude women and children and they boil the meat in herbs for a long time and eat*,*”* Female discussant, Litein-4, riverine zone.

Two ant species, *“kilik” (Messor angularis)* and *“butbutie”*, *(Crematogaster sp)* were used by the Tugen to test meat for safety of consumption by placing a piece of meat from the slaughtered animal near the ants’ nests then people would observe whether they (ants) would attempt to eat it or not. If the ants avoided the meat it was considered unsafe for consumption but if they did not, the meat was considered harmless hence eaten as exemplified in the following excerpts.

““*Kilik*” *are ants that are used to test the safety of meat from dead animals…*. *When they eat the meat and die*, *the meat is not safe and when they eat and they survive then the meat is safe for consumption”*. Female discussant, Kipcherere-1, midland zone

*“You know there are methods the old men used traditionally*. *They cut a small piece of the meat and take it to the “butbutie”*. *If they eat there is no problem*, *people eat*. *If they don’t eat people leave [the meat]”*. Male discussant, Litein-1, riverine zone

Another method was burying the spleen in soil and if it appeared to increase in size the meat was considered unsafe for consumption but if there was no increase the meat was considered safe for consumption as demonstrated below.

*“If an animal dies*, *the old men put the spleen on the ground [covered in soil]*. *If in a short while it swells and bursts they know it is anthrax”*. Male discussant, Sirata-1, lowland zone

### Disposal of dead animals and foetuses

Community members used multiple methods to dispose of dead animals. Besides consumption, it was established in FGDs that animal cadavers were also buried whole, skinned then buried, skinned and given to dogs, skinned and thrown in the open or burned. Among survey respondents, only n = 153 (27.5%, N = 556) and n = 50 (9%, N = 557) reported that they always buried or burned sick animals after death, respectively. Aborted foetuses, were always buried in n = 159 (28.4%, N = 560) of cases. Up to n = 220 (40%, N = 550) of respondents reported ever leaving foetuses out in the open to rot and n = 404 (73.2%, N = 552) feeding them to dogs. There was a statistically significant association between sex and feeding aborted foetuses to dogs (χ^2^ = 18.114, df = 4, p = 0.001) with more men inclined to engage in the practice (Table 2). Among the Ilchamus, it was a taboo to bury dead livestock as shown in the following excerpt.

*“We do not bury dead livestock like human beings*. *You just slaughter the animal because it is also a taboo not to cut open the abdomen of a dead animal even if it will be fed to dogs”*. Female discussant, Salabani-2, lowland zone

Even when they resolved to bury the dead animals, some community members would skin the animal because of the belief that *“when it [a sick animal] is buried with the skin/hide on*, *it will cause harm to the remaining stock and they might die*,*”* as reported by a female focus group discussant from Borowonin, the highland zone.

### Management of livestock diseases

Management of livestock diseases was mainly left to community members and was traditionally prescribed for men. Only n = 242 (44%, N = 550) of respondents relied on a veterinary officer to treat their sick livestock most of the time whereas n = 339 (61.6%, N = 550), mostly bought veterinary medicines and treated the sick animals without the guidance of a veterinary officer. When animals aborted, n = 364 (65.7%, N = 554) of respondents often treated them with conventional veterinary medicines while n = 125 (22.6%, N = 553) used herbal treatments. Veterinary services were considered expensive most of the time by n = 343 (63.9%, N = 537) of respondents. An equal proportion of respondents n = 468 (84%, N = 557) reported often handling sick livestock and assisting deliveries with bare hands. Men were more prone to handling sick livestock with bare hands (χ^2^ = 11.185, df = 4, p = 0.025); treating sick livestock without consulting a veterinary officer (χ^2^ = 18.326, df = 4, p<0.001); consider veterinary services as expensive (χ^2^ = 13.210, df = 4, p = 0.010); and reporting that accessing veterinary medicines was difficult (χ^2^ = 32.627, df = 4, p<0.001); probably due to their experience in managing livestock diseases (Table 2). On the other hand, more women were inclined to seek a veterinary officer’s services for livestock treatment (χ^2^ = 17.539, df = 4, p = 0.002); think that veterinary services were easy to access (χ^2^ = 33.915, df = 4, p<0.001) but find it difficult to administer veterinary medicines (χ^2^ = 26.884, df = 4, p<0.001) probably because traditionally, the role of livestock disease management was not theirs (Table 2).

## Discussion

The occurrence and coverage of RVF is determined by a multiplicity of factors which include availability of susceptible hosts, competent vectors, adequate precipitation and permissive ecology besides human behavior [18, 20, 33]. The current study found that livestock farming was ranked second in importance as a livelihood activity after crop farming and farmers kept cattle, sheep and goats which are susceptible to the RVF virus.

The level of knowledge of RVF signs and symptoms in humans was low in the current study. Fever was the most known to respondents and impaired vision the least. In contrast, a study on RVF knowledge, attitudes and practices in Kilombero and Kongwa regions of Tanzania found that hemorrhage was the most known sign while joint pains/jaundice were the least known [34]. Similar to the current study, the level of knowledge of RVF in Tanzania was equally low. The disease mainly manifests as an uncomplicated febrile illness with flu-like symptoms but may involve hemorrhage, encephalitis or visual impairment [3] in <8% of cases [23]. Thus, the paucity in knowledge of RVF signs and symptoms may lead to misdiagnosis implicating other febrile ailments, such as malaria, that may be endemic in a region [34]. Inhabitants of Baringo County have been found to be knowledgeable of malaria signs the symptoms hence their ability to relate its symptomatology with that of RVF [35]. Unlike the Somalis in North Eastern Kenya who had a local name, “*sandik”* (bloody nose), for RVF, [26], neither the Tugen nor Ilchamus had a widely accepted term possibly because the disease was reported for the first time in 2006–2007 [17].

Good knowledge of possible RVF transmission routes was statistically associated with low risk of exposure to the RVF virus in the binary logistic regression. This finding concurs with another study on RVF knowledge, attitudes and practices in Ijara-North Eastern Kenya, where high knowledge of preventive measures was associated with high knowledge of RVF [26]. The study further showed that high knowledge of the disease was not associated with age, sex, education, marital status, household size [26]. Another study in Kilombero and Kongwa regions in Tanzania found that besides being male or coming from Kongwa, other socio-demographic characteristics had no effect on knowledge on RVF transmission, symptoms and prevention [34]. Another RVF study in Mayotte reported that having none or primary education increased the risk of RVF infections [22]. This shows that knowledge of RVF can be determined by factors which vary from place to place. In Baringo, the difference in knowledge of risk practices between the midland, lowland and riverine zone may have been as a result of differential exposure to livestock diseases and access to veterinary services.

That only few of the survey respondents knew that mosquitoes could transmit other (unspecified) diseases besides malaria was indicative of a knowledge gap. The uncertainty of the cause of RVF was further established through FGDs in which discussants implicated bad air, monkeys, tsetse flies and rain. While heavy rain is a trigger of multiplication of RVF vectors, it is not the cause. Notably, RVF outbreaks in Kenya have been associated with the El-Nino Southern Oscillation (ENSO) phenomenon which causes higher than normal rainfall, which subsequently provides ample breeding sites for RVF vectors [36].

Communities in Baringo County did engage in risky practices through handling and consumption of livestock products, management of sick animals, disposal of foetuses and dead mature stock. The proportion of people that consumed boiled milk in Baringo County was higher than reported in other studies in Africa. For example, in Sudan, a majority consumed boiled milk, while a relatively low number consumed raw milk, fermented or cooked/made into cheese in decreasing order [37]. In Ghana, most herders drank raw milk, followed by those who consumed boiled milk while those who consumed either raw or boiled milk were the least [38]. Among pastoral communities in Ijara, Kenya, only a few people consumed boiled milk [26]. The high adoption of the practice of boiling milk before consumption may have been as a result of health campaigns against Brucellosis that were reported in the region. Milking [39] and consuming raw milk from infected animals [40] have previously been identified as possible RVF virus transmission routes.

The utilization of blood as food was part of the Tugen and Ilchamus culture and was still practiced by some. The practice has been associated with pastoral communities who hold the belief that raw blood is nutritious [27]. However, contact with blood has been implicated as a risk factor of RVF virus infection during outbreaks in South Africa [21] and Kenya during the 2006–2007 [1] and 1997–1998 outbreaks [25]. Blood has been identified as part of body fluids that are highly viraemic hence very infective [39].

The use of observation, ants, herbs or the spleen to determine meat safety reflected the role indigenous knowledge played in determining community health outcomes and suggests the need for further research into the efficacy of these methods. Limited uptake of meat inspection after domestic slaughter showed that there was risk of consuming infected meat in the event of an RVF outbreak. During the 2006–2007 outbreak, the disease was transmitted to humans from infected animals through slaughtering, skinning and consumption of infected meat [17].

The use of livestock products as part of treatment courses carries potential for exposing sick people and their care givers to infection during an RVF outbreak since they will slaughter, prepare or consume animal products as exemplified in this study. Similar practices have been recorded during RVF outbreaks. For instance, fat extracted from mutton (which is derived from a highly susceptible animal to RVF), has been used in treatment of RVF symptoms like fever and hematochezia [27]. Raw blood, unpasteurized milk and fat derived from sheep have also been used in treatment of people with RVF in Ijara [28].

The processes through which foetal material are disposed can increase risk to RVF in an outbreak setting since birthing fluids contain high volumes of the infective virus [39]. Indeed, a study of RVF sero-positivity in northern Kenya conducted after the 1997–1998 outbreak established a statistical association between RVF sero-positivity and disposal of aborted foetuses [40]. In Mayotte, sero-positivity was associated with aiding livestock in delivery and contact with/disposal of aborted foetal material [22]. In this study, aborted foetuses were not always burned or buried indicating that poor disposal was a risk factor in the area.

Carcass disposal in the study site was mainly by consumption or burying. Among the Ilchamus, it was against the culture to bury a dead animal and consumption was preferred. This practice was found to be further reinforced by the belief that boiled meat was safe for consumption and carried potential for challenging the regulations provided for disposal of condemned carcasses. A similar belief was noted in Ijara, where people reported that boiled meat carried no disease [28]. In Tanzania, a study on RVF found that survey respondents skinned dead animals, buried or left it in the open [34].

Cattle, sheep and goat diseases were mainly managed by men using herbal and conventional medicines without the guidance of veterinary officers in Baringo. In addition, sick livestock were often handled with bare hands. Combined, these risky practices enhanced possibility of infection during an outbreak. Use of protective gear was also found to be a challenge in Tanzania, where only a quarter of respondents reported using them in handling dead animals [34]. The current study suggests that in the event of an outbreak, men and women would be exposed differentially, with men being at higher risk than women due to their role in treatment of sick animals and slaughtering. Similarly, Anyangu [1] and Nguku [15], reported that men were more at risk occasioned by animal related exposures through herding, slaughtering, skinning and milking of livestock during the 2006–2007 RVF outbreak in Kenya. An earlier study in Ijara district, in northern Kenya, conducted after the 1997–98 outbreak found that men had a three times more likelihood of sero-positivity compared to women due to exposure to infected vectors and animals [40]. Seufi [41], reinforced this outcome from the 2007 outbreak in Sudan that found that males aged between 15–19 years were most susceptible compared to women. Occupations such as being a farmer or housewife were also found to put individuals at risk [41]. In Mayotte, being male has been associated with RVF virus sero-positivity because men spent longer periods outdoors facilitating exposure to infected mosquitoes [22]. Thus, the role of gendered division of labour needs consideration for effective RVF risk management.

### Conclusion

Communities in Baringo County were found to have limited knowledge on RVF causes, human signs and symptoms. Poor handling and consumption of livestock products, treatment of livestock, disposal of foetuses and carcasses were identified as possible routes of exposure to RVF virus in Baringo County. Men and women would be differentially exposed to the disease based on their gender roles in livestock farming.

The study underscores the importance of qualitative data in understanding community knowledge, attitudes and practices on diseases. In this study, it is through focus group discussions that beliefs and practices that would endanger lives such as taboos on burying dead livestock, skinning or cutting open an animal’s abdomen before disposal; traditional methods of checking for meat safety and using animal products in disease management were identified as RVF risk factors. This demonstrates that exclusive use of quantitative methods of data collection in behavioral studies can lead to loss of opportunity to gather critical insights into social problems.

The study recommends that community members should be consistently provided with information on RVF seasonality, manifestation in humans and livestock and the risk factors to strengthen their capacity in engaging in participatory disease surveillance and prevention. The health information should be tailored specifically for the local context to constructively challenge the existing myths and misconceptions. It should be relayed orally, preferably in local languages so that both the literate and illiterate community members understand. This is particularly important since loss of knowledge on RVF is possible owing to lengthy intervals between outbreaks. In addition, veterinary services within the county need to be made more accessible and affordable for effective livestock disease control.

### Limitations of the study

This study was conducted nearly a decade since the first reported RVF outbreak in Baringo County. Therefore, it focused more on assessment of current knowledge and risk practices than practices conducted during the last outbreak. Adherence to good practice in livestock production and RVF control was self-reported by the study participants rather than observed by the researchers. Therefore, there is possibility that knowledgeable respondents may have stated that they engage in good practices because they know it is desirable. While qualitative data was collected through focus group discussions, the findings are very specific to the Baringo County context and cannot be generalized to other areas. These limitations notwithstanding, the study provides insights into the risk factors that would expose the community to RVF in the event of an outbreak.

## Supporting information

S1 Info Data for RVF paper. (XLS)

S2 Info RVF Focus group discussion guide. (DOCX)

S3 Info Checklist STROBE checklist. (DOC)
