# Supplementary material for: “We do not bury dead livestock like human beings”: Community behaviors and risk of Rift Valley Fever virus infection in Baringo County, Kenya
Source: PLoS Negl Trop Dis. 2017 May 24;11(5):e0005582. doi: 10.1371/journal.pntd.0005582 (PMC5460880; doi:10.1371/journal.pntd.0005582)
Supplement: S2 Info — (DOCX) [file pntd.0005582.s002.docx]

***RVF FGD Schedule***

1. What are the types of livestock assets owned in this community? (Probe for type, proportions and factors that determine the kind and quantity of livestock a household and community can own)
2. As per category, identify the various uses of livestock in this community (Probe for food sources- meat, milk and blood, drought power, raw materials (dung, skins, hides and bones), manure, social and cultural identity, stores of food and wealth, mediums of exchange and sources of cash)
3. Explain the distribution of livestock production roles among men, women, boys and girls in this community as per livestock category (Probe for grazing of mature and young livestock, watering, taking care of sick, injured and gestating animals, sourcing for treatment, construction of livestock sheds, livestock protection from predators and theft and average time spent on these activities per day and season)
4. As per livestock category, identify important livestock diseases especially RVF (Probe for local/popular/scientific names, rank diseases in order of importance, seasons of occurrence, how farmers identify disease symptoms, actions taken for each disease whether preventive or curative, disposal of sick and dead animals, meat inspection -using veterinary care or alternative treatments such as herbs at household level, interventions by the government and NGOs and their reasons for these actions, whether the interventions have been effective and what needs to be done to enable the community to control and manage these diseases, impacts of diseases in this community and on livestock production)
